# Assessing the nonlinear impact of green space exposure on psychological stress perception using machine learning and street view images

**DOI:** 10.3389/fpubh.2024.1402536

**Published:** 2024-09-18

**Authors:** Tianlin Zhang, Lei Wang, Yazhuo Zhang, Yike Hu, Wenzheng Zhang

**Affiliations:** ^1^School of Architecture, Tianjin University, Tianjin, China; ^2^School of Civil Engineering, Tianjin University, Tianjin, China

**Keywords:** urban greening, street view, human perception, health planning, sustainable environment

## Abstract

**Introduction:**

Urban green space (GS) exposure is recognized as a nature-based strategy for addressing urban challenges. However, the stress relieving effects and mechanisms of GS exposure are yet to be fully explored. The development of machine learning and street view images offers a method for large-scale measurement and precise empirical analysis.

**Methods:**

This study focuses on the central area of Shanghai, examining the complex effects of GS exposure on psychological stress perception. By constructing a multidimensional psychological stress perception scale and integrating machine learning algorithms with extensive street view images data, we successfully developed a framework for measuring urban stress perception. Using the scores from the psychological stress perception scale provided by volunteers as labeled data, we predicted the psychological stress perception in Shanghai's central urban area through the Support Vector Machine (SVM) algorithm. Additionally, this study employed the interpretable machine learning model eXtreme Gradient Boosting (XGBoost) algorithm to reveal the nonlinear relationship between GS exposure and residents' psychological stress.

**Results:**

Results indicate that the GS exposure in central Shanghai is generally low, with significant spatial heterogeneity. GS exposure has a positive impact on reducing residents' psychological stress. However, this effect has a threshold; when GS exposure exceeds 0.35, its impact on stress perception gradually diminishes.

**Discussion:**

We recommend combining the threshold of stress perception with GS exposure to identify urban spaces, thereby guiding precise strategies for enhancing GS. This research not only demonstrates the complex mitigating effect of GS exposure on psychological stress perception but also emphasizes the importance of considering the “dose-effect” of it in urban planning and construction. Based on open-source data, the framework and methods developed in this study have the potential to be applied in different urban environments, thus providing more comprehensive support for future urban planning.

## 1 Introduction

Cities, as vital components of contemporary society, serve as central hubs for social, economic, political, and cultural activities (1, 2). Their intricate spatial structures, diverse socio-economic activities, and intricate interplay between human and natural environmental factors render them unique types of environments, exerting significant impacts on urban residents (3). Within the urban fabric, green spaces (GSs), easily accessible natural elements in daily life, hold indispensable importance for city dwellers (4–6). Its offer various social, environmental, and ecological benefits, such as mitigating urban heat island effects (7), enhancing urban hydrological cycles (8), and increasing biodiversity (9). However, the significance of GSs extends beyond these factors. Increasing research indicates that GS exposure positively influences the mental health of urban residents (10, 11).

GS exposure is commonly conceptualized as the extent of individual or group interaction with natural environments (12), and it plays a significant role in the physical and mental wellbeing of residents (13). On the one hand, GS exposure can improve residents' health by promoting outdoor activities (14, 15). On the other hand, it can directly alleviate mental fatigue and improve negative moods (16), and even enhance psychological states at a cognitive level (17). The effects of GS exposure arise from either direct or indirect sensory stimuli, including aspects such as color, shape, sound, and smell (18). In this process, visual perception is the most significant source of sensory exposure to GSs, with the most notable impact on psychology (19). Compared to traditional methods based on the normalized difference vegetation index (NDVI) (20) or land cover (21), this visibility-based evaluation method is more advantageous in research on the benefits of GS exposure (22, 23).

Theoretically, the positive effects of GS exposure on mental health have been recognized (24), but our understanding of its mechanisms remains incomplete. On one hand, most studies are based on subjective and indirect measures, such as subjective evaluations, the distance from the community to urban GSs, and statistics on GSs surrounding communities. These measures do not provide detailed information on actual psychological perception or any direct psychological perception information (25). This process is often limited by the collection of urban stress perception and the precise spatial correspondence between GS exposure and stress perception. On the other hand, most studies focus solely on linear impact results, ignoring the complex, non-linear impact processes. This hinders our exploration of the mechanisms through which GS exposure affects mental health, resulting in research findings that cannot be effectively applied in actual urban planning and construction (18).

Traditional psychological research often employs questionnaire surveys to assess stress perception in individuals and groups (26, 27). Although these methods have yielded certain research outcomes, the data collection process often consumes a considerable amount of time and human resources. Moreover, such methods are highly dependent on the survey subjects' memory and descriptive abilities, which may introduce errors (28). Therefore, their effectiveness and scalability are limited. With technological advancements, the development of devices such as eye trackers and electroencephalograms allows researchers to directly measure physiological responses, thus obtaining more accurate data on stress perception (29, 30). However, these devices are costly and complex to operate, making them unsuitable for large-scale research (31–33).

The combination of machine learning and street view images (SVI) offers new perspectives and methods for urban psychological perception research. This approach can help us identify spatial characteristics on a large scale at the urban level (34) and capture the complex correlations of micro-level features (35). It enables a direct spatial correspondence between urban environmental features and residents' perception characteristics. The method of evaluating street environment characteristics and human perceptions through street view images is highly accurate in capturing wide-ranging environmental features and perceptions (36). For instance, Ki et al. (37) analyzed the impact of GS exposure on walking time in community streets, and Li et al. (38) explored the relationship between the urban built environment and residents' health status. Further research has delved into predicting built environment characteristics (39), measuring human perceptions (40), assessing urban environmental stress (41), evaluating urban safety (42), and measuring the restorative qualities of campus environments (43).

Although machine learning-based methods for psychological perception measurement have been applied in other related studies (44, 45), existing research often estimates psychological perception based on a single, direct question (40, 45, 46). While the single-question measurement approach is easy to operate and reduces the burden on respondents, thereby improving the efficiency of perception measurement experiments, it oversimplifies the evaluation process of psychological stress perception. This simplification results in outcomes that fail to fully capture the complex, multidimensional psychological responses of volunteers when faced with street view scenarios (47). Studies have shown that when addressing symptoms such as stress, depression, and anxiety, the inadequacies of single-item measures in fully capturing the complexity of psychological constructs become more evident (48). Furthermore, this approach is more susceptible to social desirability bias or recall bias, thereby affecting the accuracy of the results (49). This has led to widespread skepticism about the street view perception evaluation method among questionnaire users. Therefore, it is necessary to construct a multidimensional psychological stress perception evaluation framework to compensate for the shortcomings of existing evaluation methods. This framework will allow for the extraction of psychological stress perceptions from multiple dimensions and their application in large-scale psychological stress perception assessments.

Building on previous research, this study makes further explorations in the following three areas: (1) Utilizing SVI to accurately measure urban GS exposure and psychological stress perception, ensuring spatial consistency and providing direct evidence of their relationship. This demonstrates the potential of using open data for large-scale psychological perception research; (2) Combining interdisciplinary theoretical knowledge to construct and validate a multidimensional stress perception evaluation framework, revealing the intrinsic formation mechanisms of environmental stress perception. This method can be conveniently applied to studies in other cities, providing a powerful tool and reference for related research; (3) Using interpretable machine learning algorithms to deeply uncover the complex, non-linear effects of GS exposure on psychological stress perception and identify its thresholds. This emphasizes the need to consider the “dose-effect“ of GS exposure in urban planning and construction, seeking the optimal level of GS layout to effectively enhance spatial quality.

This study focused on the central area of Shanghai and collected data from Baidu SVI to perform semantic segmentation. These segmented images served as the basis for extracting indicators related to urban GS exposure. Subsequently, utilizing a machine learning model based on the Support Vector Machine (SVM) architecture and integrating stress perception scales, we achieved spatial distribution measurement of psychological stress perception. Ultimately, by conducting regression analysis with a machine learning model based on the eXtreme Gradient Boosting (XGBoost) algorithm, we uncovered the nonlinear relationship between GS exposure and residents' psychological stress perception in urban environments. This method of assessing street environmental characteristics and human perception through street-view images demonstrated high accuracy in capturing extensive environmental features and perceptions (36). The contribution of this study not only constructs a novel and effective stress perception evaluation framework for assessing the psychological stress of urban residents across a broader geographical range and at a finer granularity but also reveals the complex impact process of GS exposure on residents' stress perception. This is conducive to the formulation of government public health policies, guiding future urban planning and construction, and ultimately enhancing the wellbeing of urban residents.

## 2 Methodology

### 2.1 Research framework

**Figure 1:**
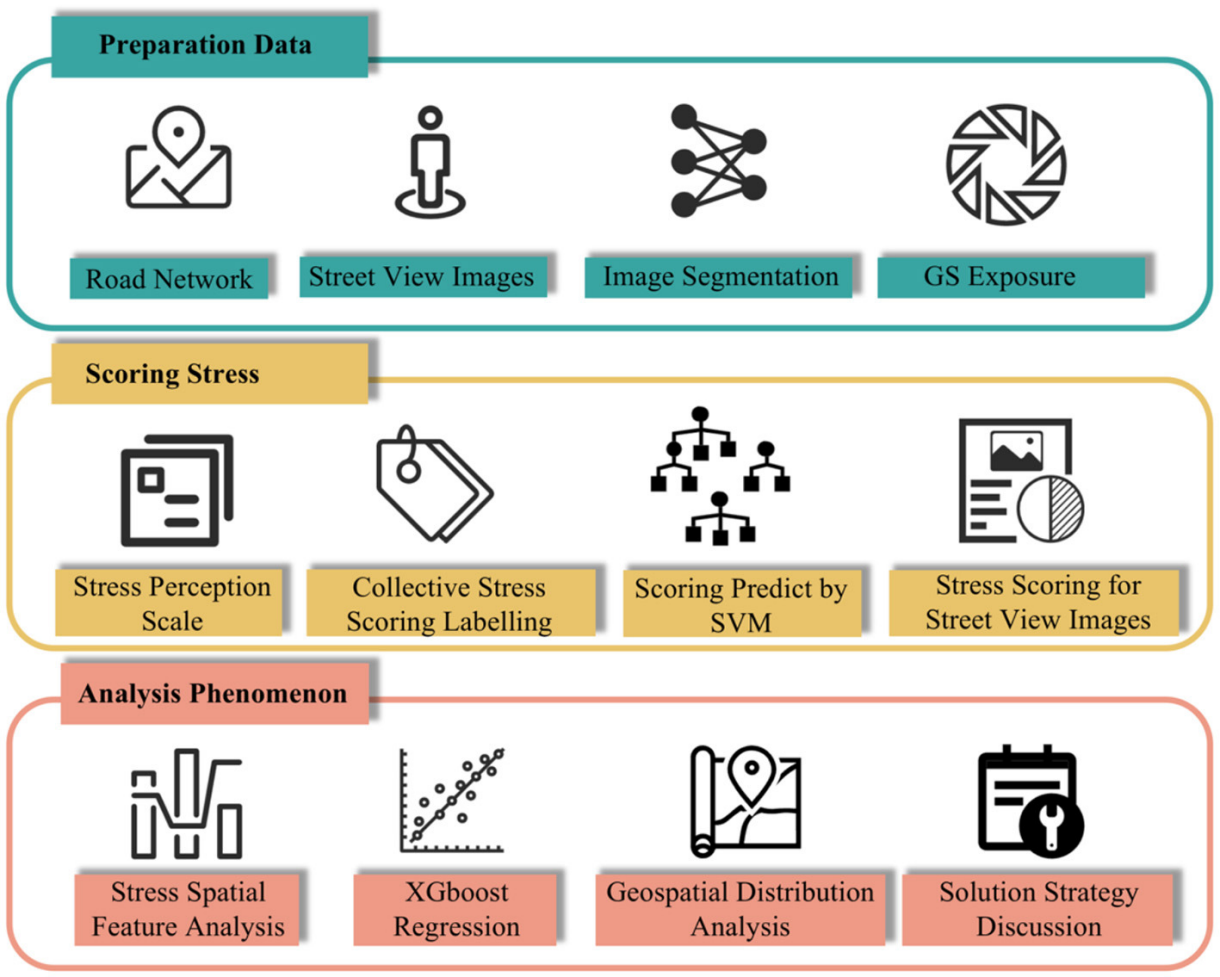
Research framework for the present study.

This study was divided into three main stages (Figure 1). First, we collected street network data from the study area using OpenStreetMap (OSM) and generated SVI sampling points at 50 m intervals. We then accessed the Baidu Map Application Programming Interface (API) to collect Baidu SVI. Utilizing an image semantic segmentation neural network model, we obtained full-element segmentation data from the Baidu SVI and extracted GS exposure features. In the second stage, we developed a framework for measuring city-wide psychological stress perception. We collected volunteers' scores using a psychological stress perception scale and created a resident psychological stress perception label dataset, which served as the training data for a machine learning model based on SVM. This enabled us to achieve city-wide measurement of resident psychological stress perception. In the third stage, the complex association between GS exposure and stress perception was explored through a machine learning model based on XGBoost, and strategies for enhancing urban GSs were proposed based on these findings.

### 2.2 Study area

**Figure 2:**
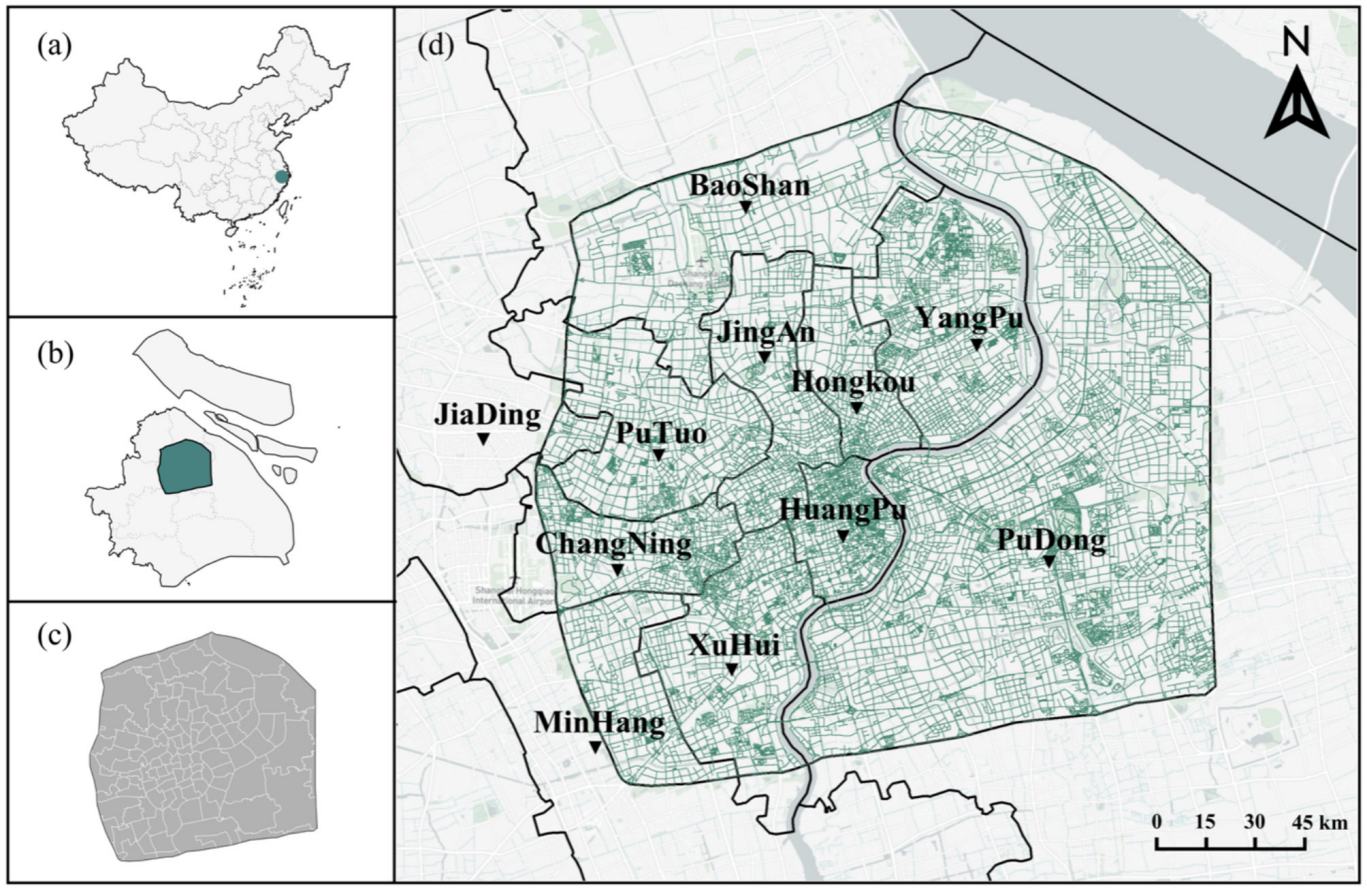
Study area. **(a)** China, **(b)** Shanghai, **(c)** Central area of Shanghai, **(d)** Road network and administrative divisions.

Shanghai is situated in the eastern part of China, between longitudes 120°51′E and 122°12′E and latitudes 30°40′N and 31°53′N. It serves as China's economic and financial center, and its level of urban development is among the highest in the country (Figure 2). The city experiences a subtropical monsoon climate, with an average temperature of 25.8°C. The focus of this study is on the central area of Shanghai, which is the region enclosed by the outer ring highway. This area includes Huangpu District, Xuhui District, Changning District, Jing'an District, Putuo District, Hongkou District, Yangpu District, and portions of Pudong New Area, Minhang District, Baoshan District, and Jiading District. Covering a total area of 660 km^2^, it is a high-density urban region characterized by intense economic and social activities, a large population, and a blend of built environments and natural elements. Additionally, this area provides abundant Baidu SVI data, which contributes to the accuracy of the study's results.

### 2.3 SVI data collection

**Figure 3:**
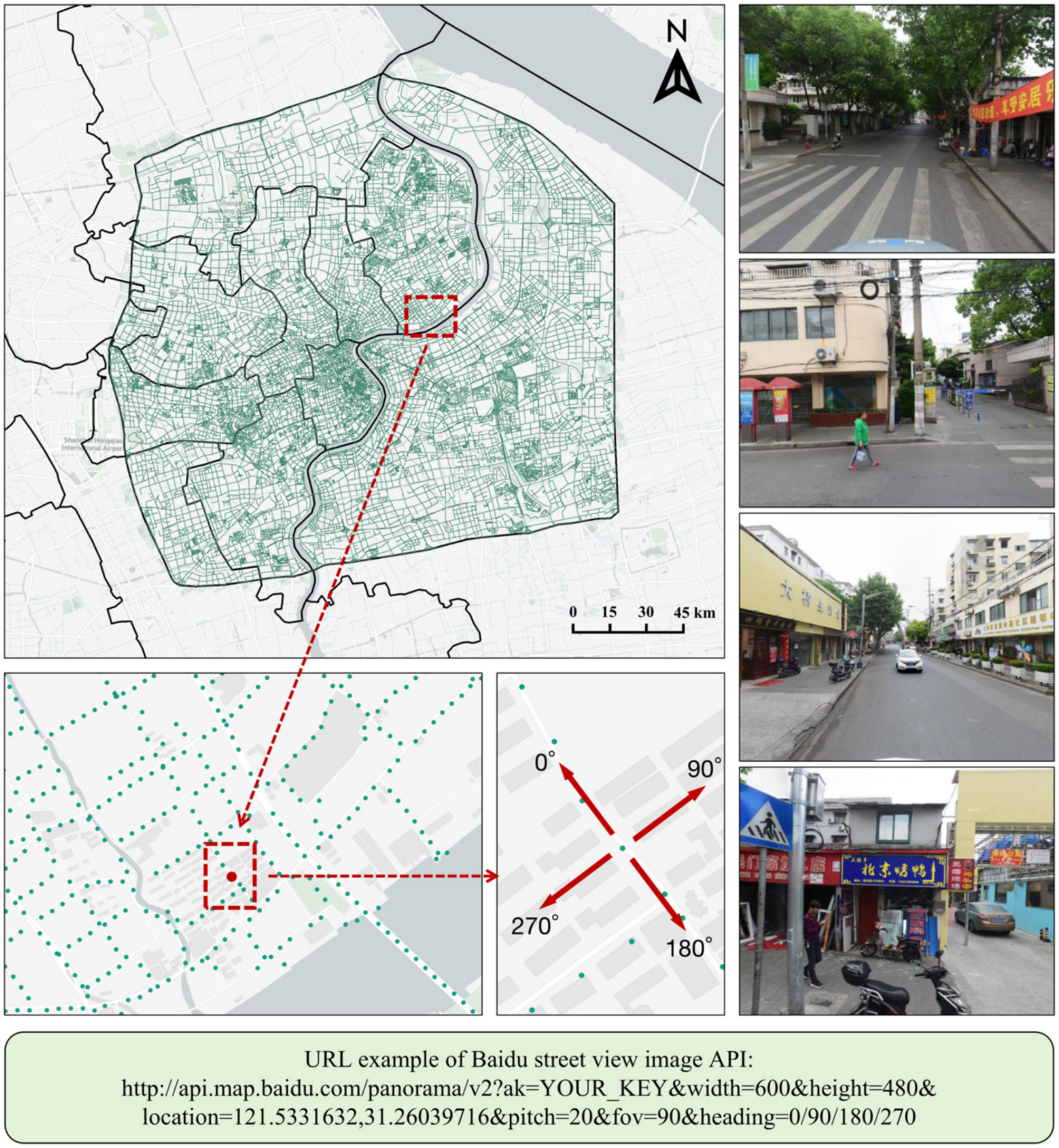
Example of the Baidu SVI collection process.

SVI are widely used in the analysis and assessment of urban environments, enabling perception and observation from a human-centric viewpoint (50). Among these platforms, Google SVI and Baidu SVI offer high-quality data and are most commonly used in research. These SVI platforms provide API, allowing users to download large-scale, quantitative data. In this study, we collected SVI from Baidu Maps (https://api.map.baidu.com/panorama/v2?ak=YOURKEY), which offers good coverage for Chinese cities (41). We generated Baidu SVI collection points every 50 meters along the road networks, which we downloaded from OSM. We calculated the viewpoint directions of the sampling points in Geographic Information System to ensure that all images aligned with the spatial direction of the road. This helps to comprehensively reflect the true urban environment. Figure 3 shows an example of a Baidu SVI sampling point. In the entire study area, we generated a total of 71,533 collection points. The image resolution was set to 600 × 480 pixels to meet the requirements of this study. To avoid affecting the overall urban environment assessment, we carried out data cleaning to remove invalid and duplicate SVI and to eliminate low-quality images with issues such as blurring, overexposure, and underexposure. In total, we collected 250,776 SVI and created 62,694 360° panoramic images by stitching them together. Despite the presence of areas where street view data could not be collected, the available data samples are sufficient to meet the research needs and maintain consistency in image features. It is worth noting that the data used in our study are cross-sectional, which has limitations in tracking changes over time.

### 2.4 Measurement of GS exposure based on scene semantic segmentation

**Figure 4:**
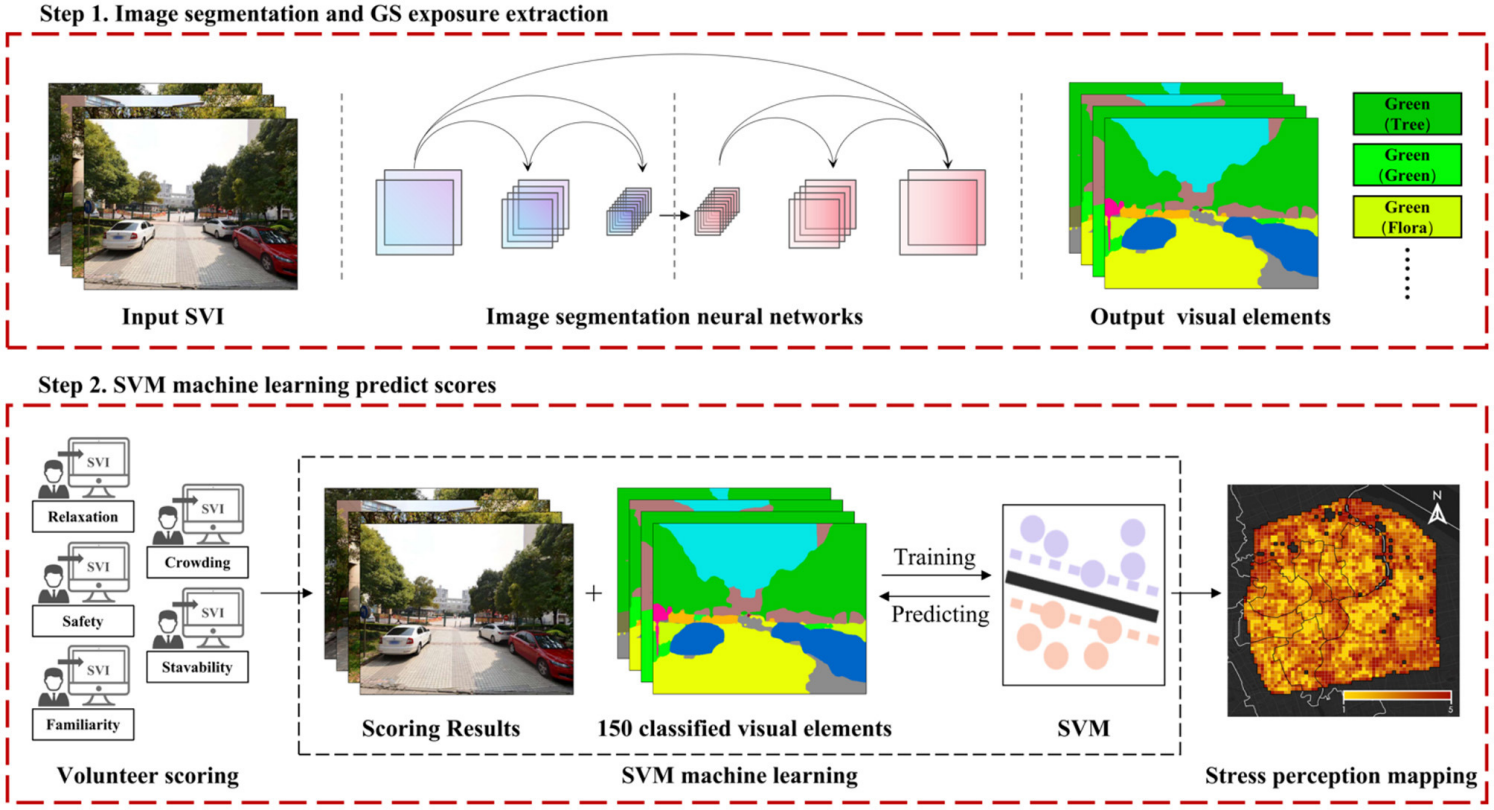
Overview of GS exposure extraction and stress perception evaluation process based on machine learning.

The scene semantic segmentation technology employed in this study exhibits robust segmentation and identification capabilities and has found extensive application in related research (34, 35, 51). In this study, a convolutional neural network (CNN) image segmentation model was trained based on the SegNet architecture, achieving precise pixel-level classification for SVI (52). The architecture of the network is primarily bifurcated into an encoder and a decoder. While the encoder is responsible for compressing and extracting object information, the decoder reconstructs the extracted semantic information to match the dimensions of the input image. Through this methodology, each pixel is categorized and represented by a color corresponding to its specific object information. The training dataset employed for this study is the ADE_20K dataset, which encompasses a diverse range of elements such as plants, sky, road, cars, and 150 other common scene elements. Previous research has confirmed the high accuracy of this dataset (53). The training process yielded an accuracy rate of 90.83% on the training set and 89.95% on the validation set, corroborating the model's efficacy in interpretative tasks related to stress perception. Visual elements pertinent to GS exposure, such as trees, grass, and flora, were extracted to compile comprehensive data daily accessible GS exposure. Figure 4 illustrates the working process.

### 2.5 Resident psychological stress perception dataset

We deployed the SVI scoring program on the Tencent Cloud server to facilitate convenient access for experimenters conducting perception scoring experiments from any computing device. Based on the previous research and machine learning requirements, we recruited a total of 32 students and staff as volunteers for this experiment. All participating volunteers were fully informed about this experiment and consented to the use of collected data for scientific research. Descriptive statistics of the volunteers' basic information are shown in Table 1. Among the volunteers, 17 were female and 15 were male, with an average age of 34.66; the ratio of local to non-local participants was 1:1. Drawing on previous research (54), we created a psychological stress perception factor scale (Table 2). This scale consisted of five factors and 12 questions, aimed at guiding the volunteers in contemplating their psychological stress perception of urban environment (55). Each volunteer received 50 pictures and was instructed to observe each image for a minimum of 30 seconds. Notably, to ensure a comprehensive coverage of the entire study area, we performed a non-systematic random selection of the SVI. We employed a 5-point Likert scale to assess the extent to which the content of each factor aligned with the volunteer's perception. We then inverted the scores for positive tendencies and calculated the psychological stress perception scores through weighted averaging.

**Table 1:** Descriptive statistics for the volunteers.

**Variables**	**Proportion/mean (SD)**
**Gender (%)**	
Male	46.87
Female	53.13
Age	34.66 (13.78)
**Education (%)**	
High school or below	25.00
College	46.87
Master degree or above	28.13
**Race (%)**	
Chinese Han	87.50
Others	12.50
**Residence (%)**	
Local resident	50.00
Non-local resident	50.00

**Table 2:** Psychological stress perception scale.

**Stress factor**	**Questions**
Relaxation	•Here you can temporarily forget the troubles in your work and life. •Here you can temporarily forget the stress caused by other people's requirements and expectations. •Here can adjust your mood and make you feel happy.
Safety	•The environment here makes you feel safety. •Crime may not occur here.
Familiarity	•The environment here is similar to your living environment. •You can integrate well into the environment here.
Crowding	•The environmental elements here make you feel crowding. •The flow of people and vehicles here makes you feel crowding.
Stayability	•You want to spend a long time here. •You are immersed in your surroundings. •You would like to visit here often.

### 2.6 Resident psychological stress perception prediction based on SVM algorithm

To investigate the psychological stress perception among urban residents, we employed a SVM-based machine learning algorithm. SVM is renowned for its effectiveness in handling high-dimensional data, providing a robust method to discover the optimal separating boundary between categories. This is crucial for accurately classifying and predicting psychological stress perception from street view image data. This particular model has been validated and has exhibited robust performance in prior studies (56, 57). In our implementation, SVI scores related to psychological stress perception served as the training data, while the features extracted from these images were used as input variables. The aggregated scores from all volunteers were compiled into an SVM training dataset. We chose the Radial Basis Function (RBF) kernel to improve the model's fit. Then, we determined the optimal hyperplane for classification by solving a convex optimization problem, as formalized in Equation 1.


(1) $$\begin{array}{l} \begin{array}{c} \text{min}\frac{\text{1}}{\text{2}}||\text{w}|{|}^{\text{2}} \end{array} \end{array}$$

Here, w is the normal vector of the decision boundary. We aim to minimize this objective function while satisfying the constraints described by Equation 2.


(2) $$\begin{array}{l} \begin{array}{c} {\text{y}}_{\text{i}}\left(\text{w}\cdot {\text{x}}_{\text{i}}+\text{b}\right)\geq \text{1} \end{array} \end{array}$$

Here, y_i_ is the class label of the sample, x_i_ is the feature vector of the sample, and b is the bias term.

For model assessment and optimization, cross-validation techniques were employed to evaluate the performance of the SVM model. During this phase, the hyperparameters of the SVM model were fine-tuned to optimize performance. A subset comprising 75% of the psychological stress perception scoring data was allocated for training purposes, while the remaining 25% served as a validation set to assess model accuracy. The SVM-based validation set achieved an accuracy rate exceeding 80%, suggesting that the model possesses robust predictive and applicative capabilities for assessing residents' perceptions of psychological stress. Figure 4 illustrates the specific operational workflow of the model.

### 2.7 Principal component analysis

Principal Component Analysis (PCA) is a prevalent method in data analysis, enabling the identification of principal features and intrinsic structures within a dataset. This study employs PCA to analyze the composition of stress perception, clarifying the contributions of various stress perception factors. The principal steps involve initially centralizing the data, followed by the computation of the covariance matrix and the determination of its eigenvalues and eigenvectors. Based on the amount of information required to be retained, the first k eigenvectors corresponding to the largest eigenvalues are selected. These eigenvectors form the new feature space. By projecting the centralized data onto the chosen eigenvectors, the dimensionality-reduced data is obtained. The computational formula for projecting into the new space can be represented as Equation 3.


(3) $$\begin{array}{l} \begin{array}{c} \text{Y}={\text{X}}^{\text{′}}{\text{P}}_{\text{k}} \end{array} \end{array}$$

Here, Y represents the matrix of the data after dimensionality reduction, *X*′ denotes the matrix of the data post-centralization, and P_k_ is the matrix composed of the selected k eigenvectors.

### 2.8 Regression analysis

To investigate the relationship between GS exposure and the psychological stress perception in the central area of Shanghai, we conducted regression analyses on the respective scores. Different regression models were employed, including the Ordinary Least Squares (OLS) regression model and machine learning models based on the XGBoost algorithm. The OLS regression model serves as a global model and estimates the coefficients of the explanatory variables in the linear equation by minimizing the sum of squared differences between the predicted and observed values in the dataset (58). The computational formula for the OLS model can be represented as follows (Equation 4):


(4) $$\begin{array}{l} \begin{array}{c} \text{Y}=\text{X}\beta +\epsilon \end{array} \end{array}$$

Here, Y is the dependent variable, X is the matrix of explanatory variables, β is the vector of coefficients, and ϵ is the vector of random error terms.

OLS is generally capable of elucidating only the simple linear associations between independent and dependent variables, falling short of analyzing more intricate variations therein. To investigate the nonlinear impact of GS exposure on stress perception, we employed a machine learning model based on the XGBoost algorithm, an optimized gradient boosting algorithm designed to address regression, classification, ranking, and user-defined prediction issues. We chose XGBoost because it can accurately interpret the nonlinearity and complex interactions between variables that are prevalent in urban planning research. Its use of a gradient boosting framework helps effectively handle various types of structured and unstructured data, improving the accuracy of exploring the nonlinear effects of GS exposure on psychological stress perception. This algorithm establishes an ensemble of decision tree models, aptly capturing nonlinear relationships between independent and dependent variables. Compared to traditional gradient boosting algorithms, XGBoost incorporates a regularization term in its objective function. The inclusion of this term aims to penalize model complexity, preventing overfitting on the training data. Specifically, the objective function of XGBoost comprises not only the prediction error but also a regularization term measuring model complexity. This regularization term is directly proportional to both the number of trees in the model and the number of leaf nodes in each tree. Thus, as the model grows more complex, the value of the regularization term increases. By balancing prediction error and model complexity in the objective function, XGBoost enhances model performance on the testing set and mitigates the risk of overfitting. We divided the input dataset into 80% and 20% for training and testing, respectively. A cross-validation method was utilized, incorporating nested Hyperopt for hyperparameter optimization, thereby refining the model's predictive accuracy (59). The objective function of XGBoost is a combination of prediction error and model complexity and can be represented as follows (Equation 5):


(5) obj(θ)=∑i=1n1(yi,y^i)+∑k=1KΩ(fk)

Here, *l* represents the loss function, delineating the discrepancy between the predicted value *y*_*i*_and the true value ŷ_*i*_. Ω is the regularization term, incorporated to impose constraints on the model, thereby averting overfitting Typically, it considers both the number of leaf nodes in the tree and the scores associated with these nodes.

## 3 Results

### 3.1 Spatial distribution characteristics of GS exposure

**Figure 5:**
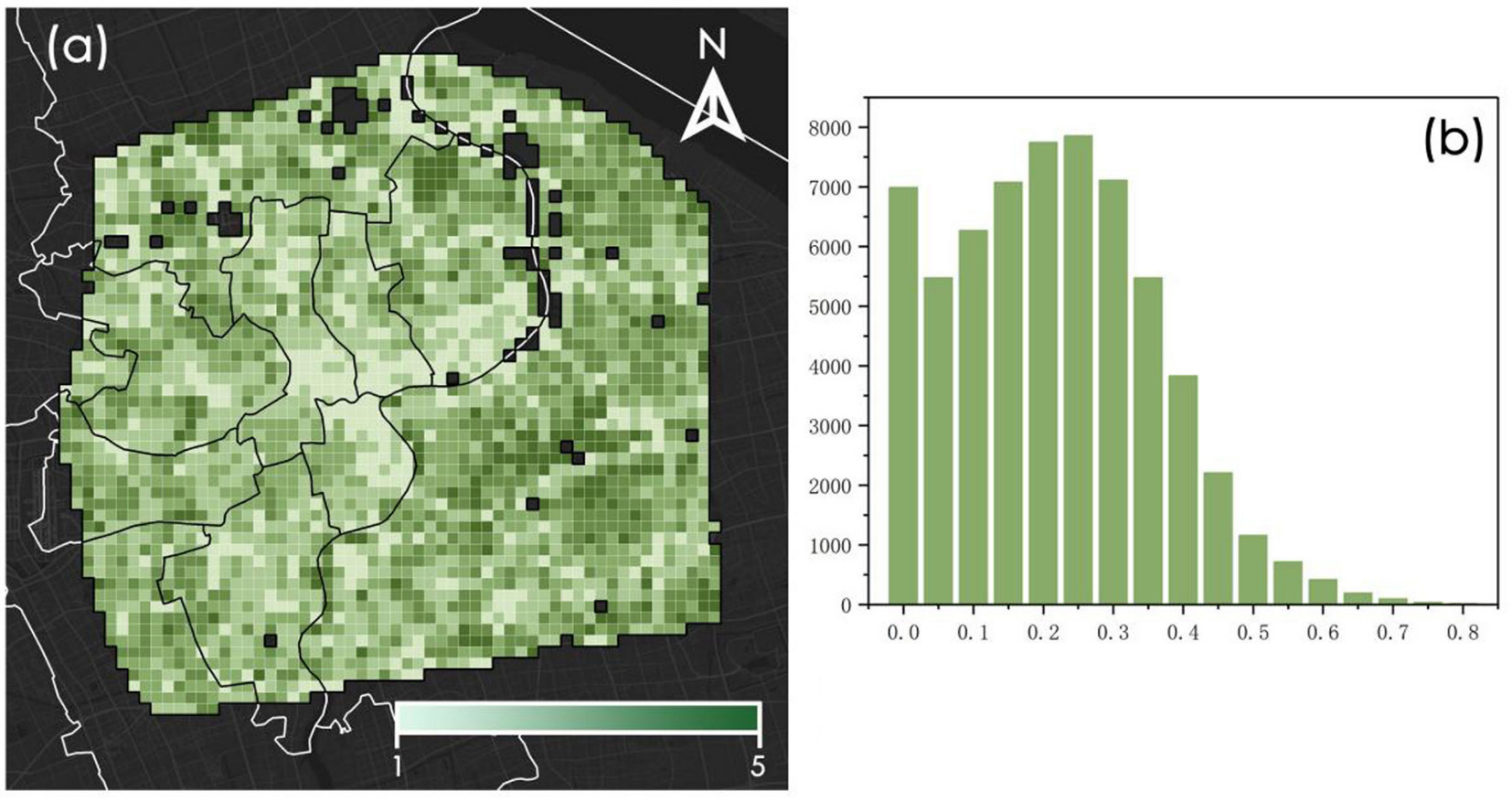
Spatial distribution and statistical distribution of GS exposure at fishnet level. **(a)** Spatial distribution of GS exposure. **(b)** Statistical distribution of GS exposure.

The results of this study are presented with reference to fishnet (500 m × 500 m) and are visualized in Figure 5. GS exposure within the central area of Shanghai varies from 0 to 0.89, with a mean value of 0.24. As depicted in Figure 5a, considerable spatial disparities in GS exposure are evident. These disparities are characterized by elevated levels of exposure in peripheral areas and diminished levels in central locations. The GS exposure on the eastern side of the research area is higher than that on the western side. This may be related to the level of urban development. Lower values of GS exposure are predominantly observed in the Jing'an District, Hongkou District, and Huangpu District, with the nadir observed at the confluence of these three districts. Conversely, elevated levels of GS exposure are primarily localized within the Pudong District. Figure 5b reveals that GS exposure at the majority of streetscape data collection points is < 0.40, indicative of a generally low prevalence of GS exposure in central Shanghai.

### 3.2 Spatial distribution characteristics of five types of psychological stress perception factors

**Figure 6:**
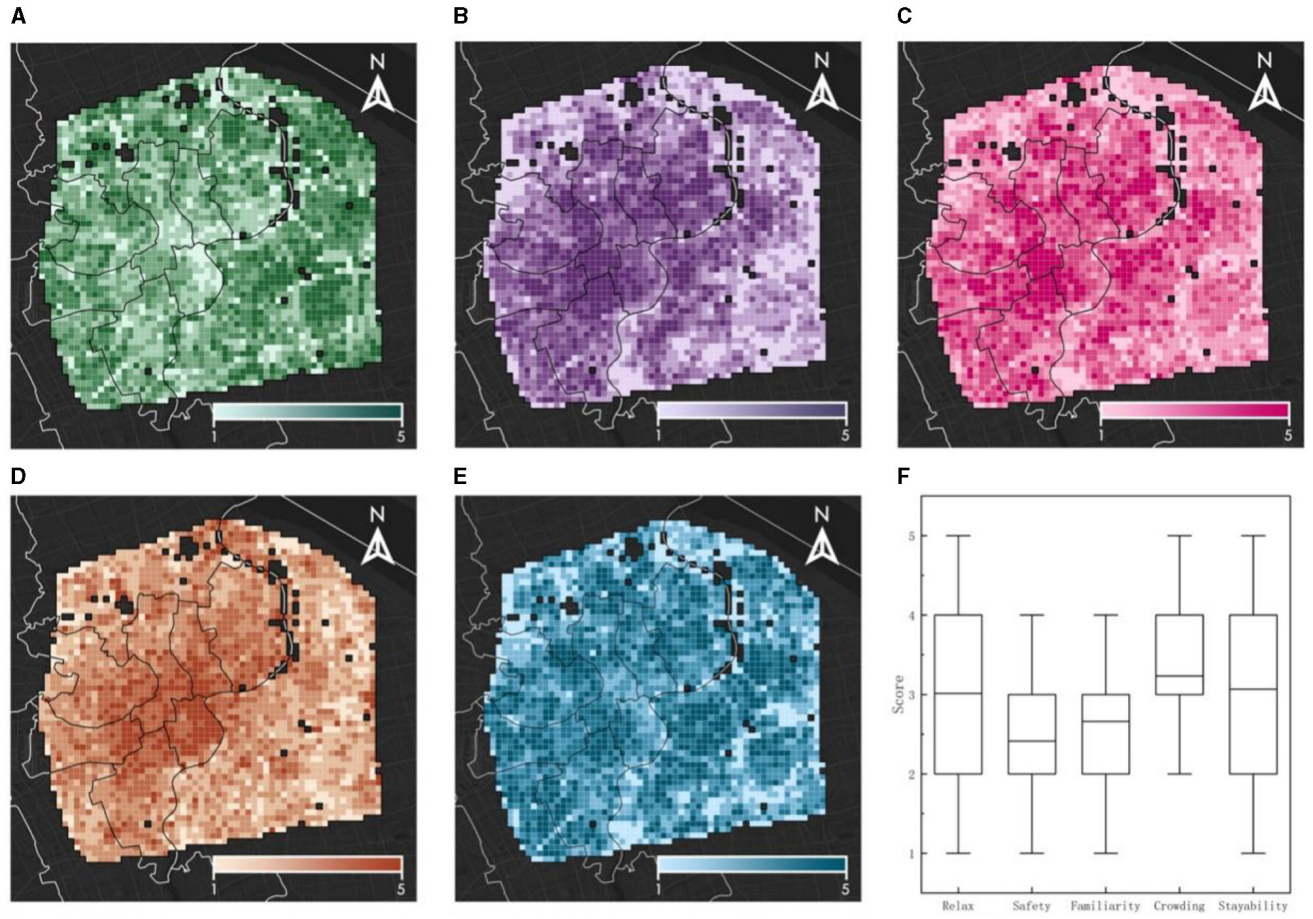
Spatial distribution characteristics and boxplot of five psychological stress perception factors. **(A)** Spatial distribution of relax perception. **(B)** Spatial distribution of safety perception. **(C)** Spatial distribution of familiarity perception. **(D)** Spatial distribution of crowding perception. **(E)** Spatial distribution of stayability perception. **(F)** Boxplot of five factors.

As illustrated in Figure 6, the spatial distribution of the relaxation factor exhibits a notable pattern, characterized by higher values at the periphery and lower values at the center of the study area. Conversely, the crowding factor demonstrates a trend of higher values in the city center and lower values toward the periphery safety and familiarity exhibit similar spatial distribution patterns, both displaying a “low-high-low” gradient from the center to the periphery. This pattern may be attributed to the increased controllability and predictability found in environments that are more familiar and easier to integrate into, which consequently elicits more positive emotional responses and encourages residents to remain in these areas. The stayability factor also manifests a “low-high-low” spatial distribution from the center outward, suggesting that residents near both the center and the periphery are less inclined to stay. This observation suggests that areas that are either highly developed or underdeveloped are equally unfavorable for residents' proclivity to linger. In summary, the average scores for relaxation and stayability are observed to be higher than those for safety and familiarity. The perception of crowding registers the highest average score, which can be associated with the built environment characteristics typical of a high-density urban setting.

### 3.3 Spatial distribution characteristics of resident psychological stress perception

**Figure 7:**
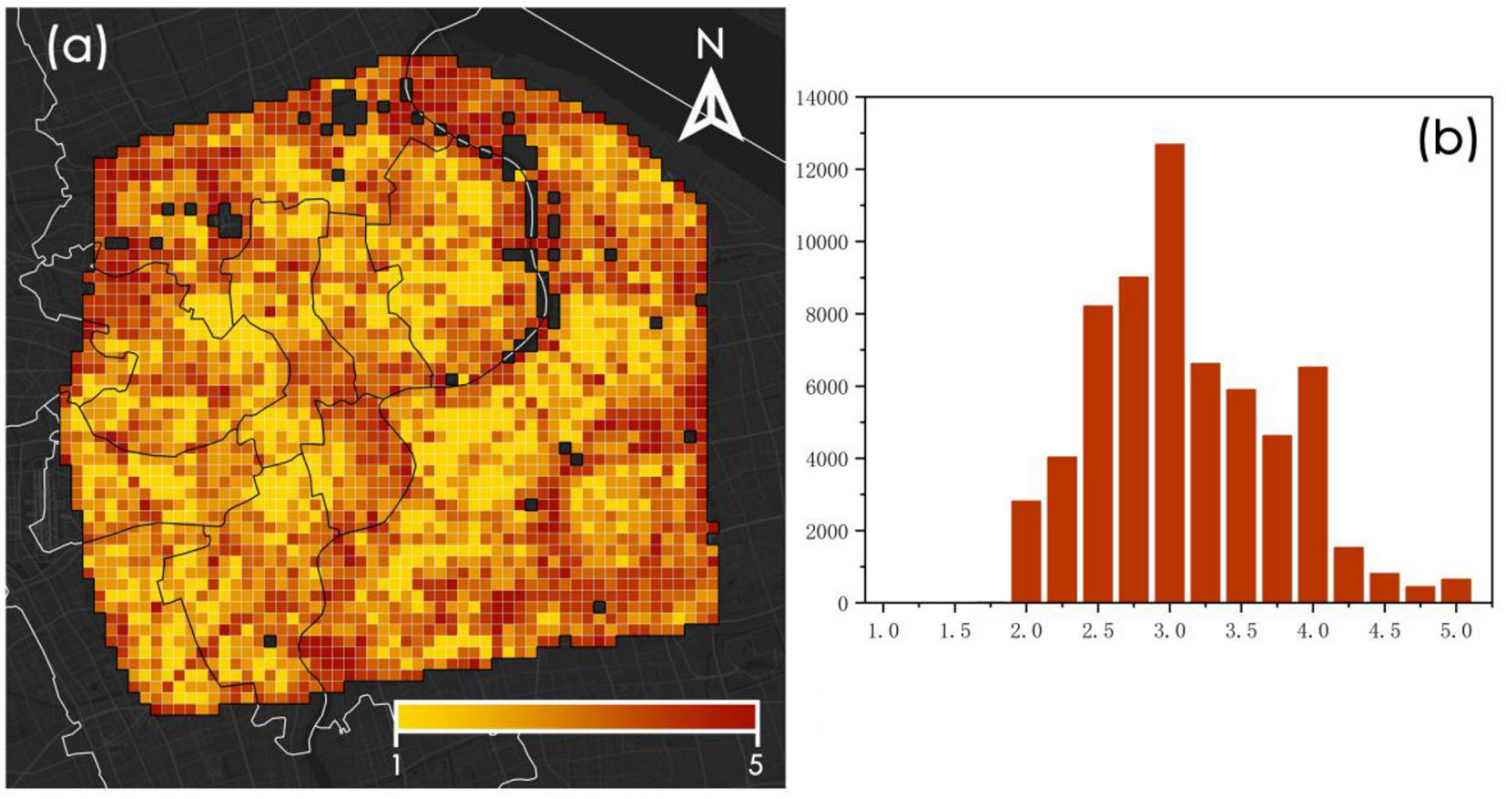
Spatial distribution and statistical distribution of psychological stress perception at fishnet level. **(a)** Spatial distribution of stress perception. **(b)** Statistical distribution of stress perception.

**Figure 8:**
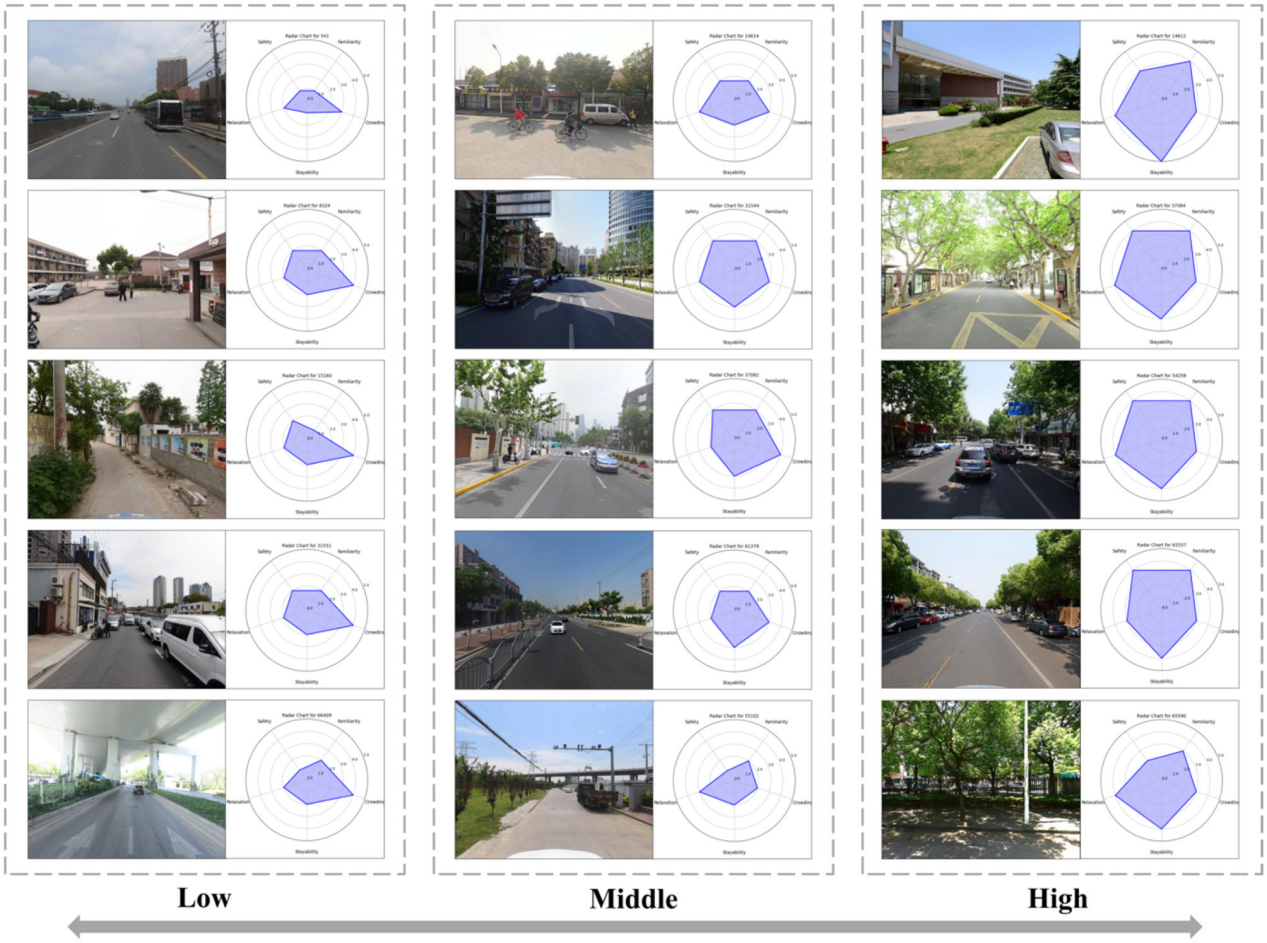
Representative SVI and its psychological stress perception factor radar map.

As depicted in Figure 7a, the spatial distribution of residents' psychological stress perception exhibits a “low-high-low” pattern radiating outward from the center. Elevated levels are primarily concentrated at the intersections of Jing'an District, Hongkou District, and Huangpu District, as well as in the periphery of the central area. Elevated levels of psychological stress perception in the central area might be attributed to its prolonged period of urban development, higher development levels, and increased building and population densities. In the periphery of the central area, higher levels of psychological stress perception can be ascribed to two principal factors: (1) Although high-quality spaces are conducive to stress relief, the more desolate scenery in the outskirts neither promotes feelings of safety nor encourages prolonged stay, thus failing to serve as an effective stress-relief environment; (2) The periphery encompasses motorways replete with a significant number of flyovers and underpasses, features that are not favorable for psychological stress relief. These factors influence residents' sense of familiarity and stayability, thereby further affecting their psychological stress perception. This suggests that both highly developed and underdeveloped areas exert a negative impact on residents' psychological wellbeing. As depicted in Figure 7b, psychological stress perception is elevated in the central region of Shanghai. Figure 8 presents representative streetscape images corresponding to high, medium, and low psychological stress perception scores.

**Figure 9:**
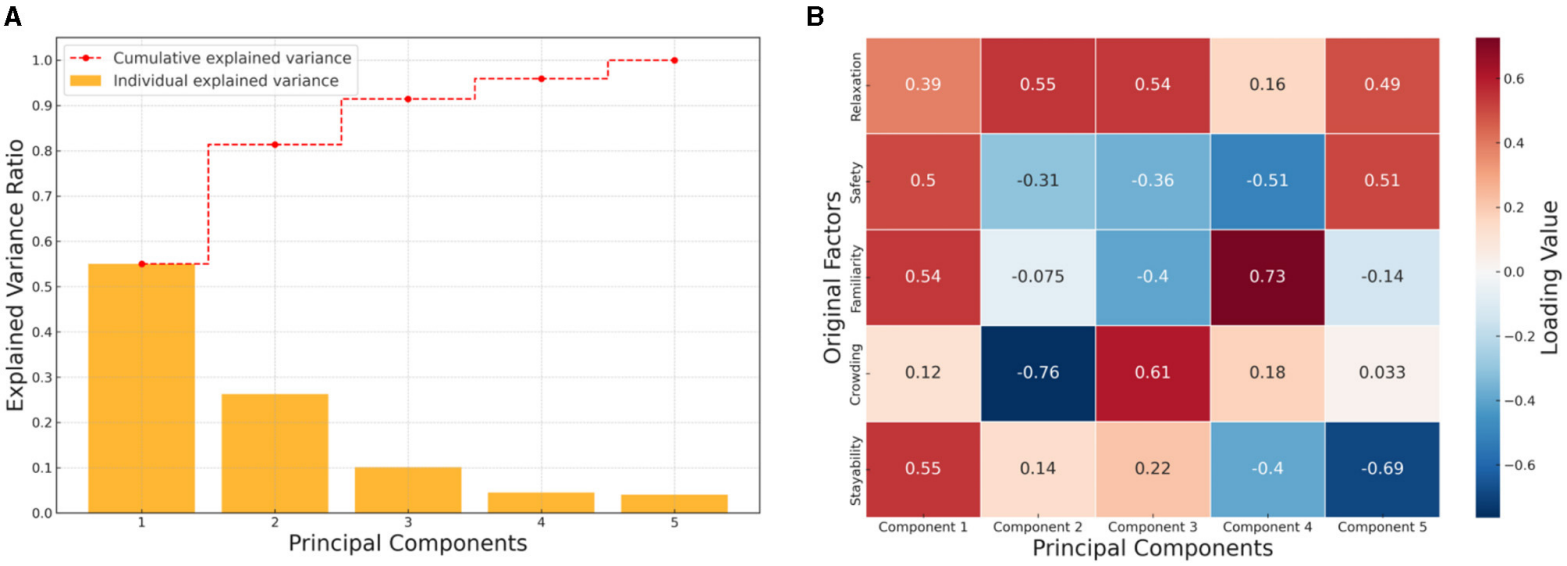
PCA of psychological stress perception. **(A)** Cumulative interpretation variance analysis. **(B)** Loading of the stress perception factor on principal components.

We employed PCA method to conduct an in-depth analysis of Stress perception in urban environments and its five constituting factors. These factors include: relaxation, safety, familiarity, crowding, and stayability. Our analysis results, as shown in Figure 9, indicate that the first two principal components collectively explain over 81% of the variance in the data. This implies that these two components have captured the majority of the information concerning residents' psychological stress perception and its factors. Specifically, the first principal component, strongly positively correlated with familiarity and stayability, accounts for 55.05% of the variance in the data. This suggests that residents' familiarity with, and willingness to stay in, an urban environment are primary factors of stress perception. An environment that is familiar and appealing could significantly reduce residents' psychological stress. The second principal component, exhibiting a strong positive correlation with relaxation and a strong negative correlation with crowding, explains 26.31% of the variance. This indicates that the sense of relaxation and crowding also play crucial roles in stress perception. In a relaxed environment, residents are likely to experience less psychological stress, whereas crowded environments may elevate their stress levels. The remaining three principal components contribute less to explaining the variance, with values of 10.09%, 4.50%, and 4.06% respectively. The relationships of these components with other factors are also more complex, possibly involving more interactions or secondary influences. Overall, our analysis has unveiled the key constituents of residents' stress perception, with familiarity, stayability, and relaxation being the predominant factors. This offers significant guidance for urban planning and design, underscoring the importance of creating an urban environment that is familiar, appealing, and relaxed.

### 3.4 Results of nonlinear regression analysis

We conducted regressions on GS exposure, perceived stress, and five perceived stress factors using both the OLS model and the XGBoost algorithm. Table 3 presents the regression performance of the two models. The results indicate that the XGBoost algorithm outperforms the OLS model in terms of goodness of fit (R^2^) in all regressions, with lower mean square error. This suggests that compared to traditional models, machine learning models based on the XGBoost algorithm exhibit higher accuracy. GS exposure explains the perception of relaxation to the greatest extent (R^2^ = 0.722), and also has a relatively high explanatory power for the perception of Stress and Stayability. However, it has a lower degree of explanation for safety, familiarity, and crowding. Table 4 presents detailed information on the total regression results of GS exposure on stress perception and the five stress perception factors. The results indicate that GS exposure is significantly negatively correlated with stress perception, with a mean coefficient of −2.814, demonstrating that GS exposure can effectively reduce stress. Moreover, the impact of GS exposure on relaxation perception is highly significant, with a mean coefficient of 4.800. Additionally, GS exposure significantly enhances safety perception (mean coefficient 1.384), familiarity perception (mean coefficient 2.262), and stayability perception (mean coefficient 4.951), indicating that it improves individuals' positive evaluation of the environment and their inclination to remain in it. The effects of GS exposure on stress perception and the five stress perception factors are all highly significant.

**Table 3:** Regression models parameter comparison.

	**OLS**			**XGBoost**		
	**R2**	**RMSE**	**Variance**	**R2**	**RMSE**	**Variance**
Stress	0.416	0.486	0.164	0.529	0.437	0.212
Relaxation	0.675	0.481	0.484	0.722	0.445	0.523
Safety	0.042	0.986	0.038	0.148	0.931	0.170
Familiarity	0.145	0.805	0.106	0.203	0.777	0.165
Crowding	0.017	0.791	0.009	0.036	0.783	0.042
Stayability	0.431	0.822	0.512	0.484	0.783	0.594

R^2^ represents goodness of fit, while RMSE represents the root mean square error. N = 62,694.

**Table 4:** Total regression results of GS exposure on stress perception and five stress perception factors.

**Variable**	**Mean coef**.	**Std. err**	***t***	***P* > |t|**	**[95% conf. interval]**	
Stress	−2.814	0.015	−193.391	0.000	−2.842	−2.785
Relaxation	4.800	0.015	330.942	0.000	4.772	4.829
Safety	1.384	0.030	46.242	0.000	1.325	1.443
Familiarity	2.262	0.024	93.430	0.000	2.214	2.309
Crowding	−0.672	0.024	−27.832	0.000	−0.719	−0.625
Stayability	4.951	0.025	200.805	0.000	4.902	4.999

Mean coef. represents Mean Coefficient, Std. Err represents Standard Error, and 95% conf. Interval represents 95% Confidence Interval.

**Figure 10:**
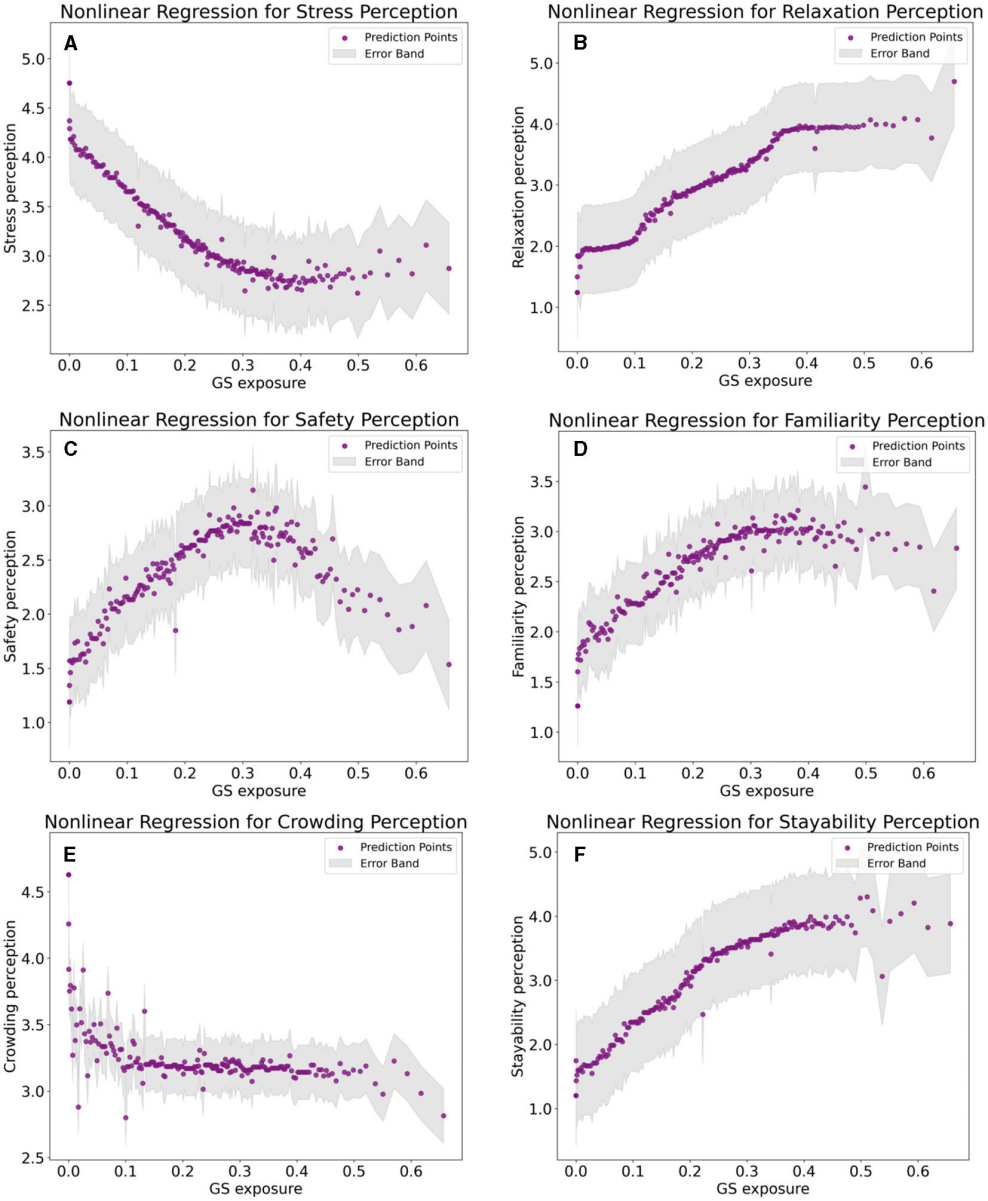
Nonlinear regression analysis. **(A)** Nonlinear regression for stress perception. **(B)** Nonlinear regression for relaxation perception. **(C)** Nonlinear regression for safety perception. **(D)** Nonlinear regression for familiarity perception. **(E)** Nonlinear regression for crowding perception. **(F)** Nonlinear regression for stayability perception.

The results of the nonlinear regression are presented in Figure 10. An increase in GS exposure results in a decrease in the stress perception, with the slope declining as GS exposure increases. Specifically, within a GS exposure range of 0–0.20, there is a pronounced and significant reducing effect; from 0.20 to 0.35, the reducing effect gradually moderates; beyond 0.35, there is scarcely any reducing effect. Thus, 0.35 can be considered the threshold at which GS exposure affects stress perception. This indicates that indiscriminately increasing GS is imprudent, and urban planning must consider the dosage of GS exposure to effectively enhance spatial quality. We further analyzed the effects of GS exposure on various stress perception factors to better understand the process by which GS exposure influences stress perception. For each stress perception factor, the impact of GS exposure varies. An increase in GS exposure will enhance relaxation perception, with the slope decreasing and approaching zero as GS exposure increases, and the threshold being approximately 0.35. The effects of increased GS exposure on safety and familiarity initially increase and then decrease, with peak values around 0.30. Notably, beyond 0.30 GS exposure, the rate of decrease for safety is much greater than for familiarity. This suggests that excessive GSs can make people feel unsafe, possibly due to obstructed sightlines. GS exposure only has an unstable alleviating effect on crowding perception in the 0–0.15 range and quickly becomes negligible. The effect of GS exposure on stayability is similar to its effect on relaxation, showing a positive increase. However, as GS exposure increases, this increasing effect gradually diminishes. Overall, the positive effects of increased GS exposure are stable and significant, but there is a threshold for these effects.

**Figure 11:**
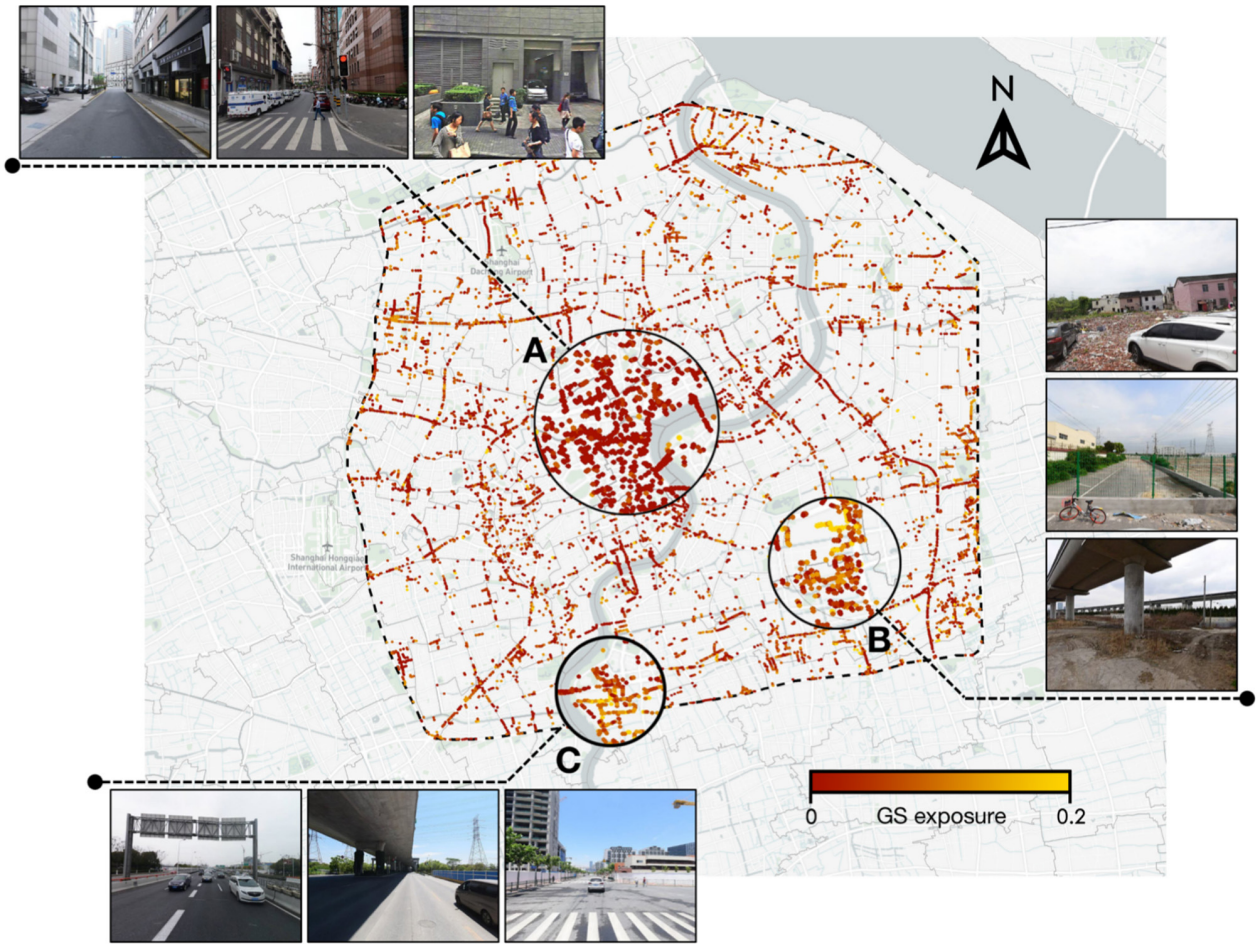
Distribution map of targeted identification for negative spaces.

Based on the above findings, to further guide urban planning and construction from a multi-dimensional perspective, we selected locations with stress perception scores above 4 (i.e., locations with high stress perception scores) and where GS exposure is < 0.20. These locations represent high-stress areas in the city, where GS exposure can have a significant positive effect. This allowed us to target areas in the city where GS exposure needs to be enhanced, to develop specific optimization strategies. In Figure 11, we display the visualization results and detailed scenarios of the targeted identification. Area A is located in the center of the study area, with a concentration of commercial enterprises and public service facilities. The area is characterized by high-rise buildings, extremely high building density, and intensive human activity, which can often cause feelings of oppression. Simultaneously, the lack of greenery in the area does not provide a psychological buffer. Area B is located in the central part of Pudong New Area. In contrast to Area A, Area B is remote, has low foot traffic, and is underdeveloped. The desolate scenery, litter, and overgrown weeds reflect a lack of development and management, resulting in a negative perceptual experience. Although this area has some greenery, its quality is low and does not alleviate stress perception. Area C is an emerging urban area under construction. Due to its development stage, the greenery is still inadequate, likely contributing to high stress perception. Additionally, the area contains numerous transportation facilities such as overpasses and highways, lacking recreational spaces, which also increases stress perception. The SVI of these areas further showcase the detailed scenarios of the regions, corroborating our observations.

## 4 Discussion

### 4.1 Urban GS exposure and psychological stress perception

The unrelenting process of urbanization has brought forth numerous urban issues (60, 61), compelling us to shift our focus toward enhancing the quality of urban spaces. Presently, the development model of cities in China has transitioned from incremental development to stock enhancement (62), with the augmentation in quantity and quality of GSs emerging as a paramount issue (15). Within urban GSs, the street or block level GS exposure has a significant impact on the quality of daily life for residents (63). Despite the widespread acknowledgment of the positive effects of GS exposure, many studies are based on small-scale samples or indirect associations, which, to some extent, limits the widespread applicability and generalizability of the research findings. Consequently, it is imperative to employ large-scale, high-precision research methods to explore in-depth the relationship between GS exposure and mental health. Our study reveals that the average GS exposure in the central urban area of Shanghai is only 23.97%, exhibiting significant spatial disparities. This indicates an issue of inequality in GS exposure in central Shanghai. We found a significant negative correlation between GS exposure and residents' psychological stress perception, which is consistent with general cognition (64). Moran's I analysis shows a significant spatial correlation between various stress perception factors and GS exposure. This finding aligns with those of Roe et al. (65), underscoring the irrefutable role of GS exposure in residents' mental health within urban spaces. High GS exposure and low stress perception exhibit spatial clustering. Notably, the impact of GS exposure is most significant on perceptions of relaxation and stayability, implying that GSs can not only provide short-term emotional relief but also contribute to long-term comfort of staying. However, the explanatory power of GS exposure is lower for perceptions of safety, familiarity, and crowding, suggesting that these factors might be influenced by multiple variables. Previous studies have only verified the stress-relieving effect of GSs without unraveling the complex process involved. Our research indicates the presence of a threshold in the impact of GS exposure, suggesting a diminishing effect on stress perception as exposure reaches a certain level. This discovery suggests that urban planning should consider the “dosage effect” of GS exposure, avoiding a blind increase in GSs and seeking an optimal level of greenery to effectively enhance spatial quality.

Although our study was conducted using the central urban area of Shanghai as a case example, which imposes geographic limitations on data collection, our findings underscore the significant role of GS exposure in reducing psychological stress. This insight can assist urban planning and public health strategies beyond Shanghai. Our novel methods and results align with other research in this field (66, 67). We also posit that the threshold effect of GS exposure on perceived psychological stress should be generally applicable, although the specific value may not necessarily be 0.35 The discovery of the GS exposure threshold provides valuable insights for urban designers and planners worldwide. It indicates that beyond a certain point, merely increasing the amount of GS may not yield additional health benefits. This finding advocates for a strategic approach to the distribution of urban GSs, focusing on optimizing layout and integration into the urban landscape to maximize public health outcomes. Our results advocate for incorporating urban GSs as a standard component of urban environmental planning, potentially influencing zoning laws, building codes, and public health policies to create environments conducive to mental health. Additionally, this study emphasizes the crucial role of quantifying psychological stress perception in understanding this relationship. By examining specific stress perception factors, we can more accurately identify and interpret the multifaceted impact of GS exposure on psychological stress perception, providing substantial academic support for further revealing the positive effects of GS exposure on improving residents' mental health.

### 4.2 Machine learning-based framework for urban psychological stress perception measurement

The measurement of stress perception helps uncover the impacts of urbanization processes on people's mental health, revealing the driving factors behind residents' psychological changes during urban development and further informing urban planning and design. Integrating urban physical built environments with citizens' psychological states within a quantifiable comparative framework is a bottleneck limiting the development of urban research (68, 69). This study developed a precise and user-friendly framework for measuring urban stress perception, with empirical measurements conducted in central Shanghai. The main advantages of the developed framework are threefold: (1) The study constructed a stress perception factor scale from a psychological perspective and integrated it into the evaluation framework. Previous research typically employed single-item questions for volunteers to directly rate the perceptions under measurement (45). However, human psychological perception is complex (70), challenging accurate measurement through a single dimension. This study incorporates psychological principles into stress perception measurement, refining it into five independent factors and twelve well-defined questions, guiding volunteers to think multifacetedly about perception, making abstract emotional issues more concrete and objective. (2) The study emphasized the importance of local volunteers. Volunteers from the same culture or region have a deeper understanding of the environment in the study area, hence providing more accurate perceptual judgments (44). Therefore, the framework developed in this study is highly accurate in characterizing the perception of Chinese cities. (3) Based on large-scale urban street view data and SVM machine learning algorithms, this study developed a framework that supports large-scale, fine-grained measurement of urban stress perception. Compared to the RF models often used in other related studies (54, 71), the SVM model exhibits better stability and interpretability in handling such problems.

This study adopted an innovative approach by measuring the impact threshold of GS exposure, fitting high psychological stress perception with low GS exposure. This enables the targeted identification of spaces in urgent need of improvement in the city, implementing GS exposure in these areas to efficiently enhance the overall spatial quality. This targeted identification method is highly accurate as it directly considers the relationship between psychological stress perception and GS exposure, ensuring the identified spaces can indeed be rapidly improved by increasing GS exposure. Additionally, this method considers the characteristics and needs of different spaces, making the implemented GS strategies more realistic and thereby enhancing the overall effect. The application of this method not only aids urban planners in more precisely identifying and improving urban spaces but also provides valuable references for future urban planning. Through this method, urban greening can be conducted more targeted, effectively elevating the quality of life and mental health levels of urban residents. More importantly, the developed framework, based on extensive open-source street view data, can be conveniently expanded to quantify the stress perception of residents in other urban areas. It is especially accurate for Chinese cities, further enhancing the practical value of the research findings and providing broader support for urban planning and decision-making.

### 4.3 Limitations and future directions

Our study has utilized high-resolution SVI to quantify urban GS exposure and psychological stress perception from a visual perspective, thereby providing direct evidence for the mitigating impact of GS exposure on psychological stress. This contribution extends the applicability of machine learning and SVI in the field of urban planning and construction. Despite these advances, our study presents several limitations that warrant consideration and offer directions for future research.

Firstly, the study primarily relies on cross-sectional data, which limits our ability to establish causality between GS exposure and psychological stress perception. This results in a shortcoming in our study regarding the consideration of temporal changes in GS exposure and psychological stress perception. Future research should consider employing longitudinal or tracking studies to uncover the underlying mechanisms positively influenced by GS exposure. Secondly, although we identified the nonlinear impact of GS exposure on stress perception based on machine learning models, there remain other unconsidered social, cultural, and economic factors that may affect psychological stress perception (72), which should be more comprehensively considered in subsequent research. Thirdly, our study employed psychological stress perception scales to quantify stress, but it did not delve into the demographic details of the volunteers. Factors such as age, gender, environmental attitudes, and professional background could bias the results and deserve further exploration in subsequent studies (73–76). Additionally, this study is based on Baidu Maps street view data, and is inevitably influenced by the limitations of Baidu Maps street view data itself. While Baidu street view images provides extensive coverage of urban areas in China, it is limited by the collection dates and update frequency, which hampers its ability to reflect seasonal and interannual changes. Furthermore, the lack of coverage of rural areas means that the framework developed in this study cannot be applied to the evaluation of psychological stress perception among rural residents.

In summary, while this study makes important contributions to our understanding of the relationship between GS exposure and psychological stress perception, there are multiple avenues for future research and refinement. These limitations offer valuable insights that can inform and guide subsequent studies.

## 5 Conclusion

The positive impact of GSs on mental health has been widely acknowledged, but there remains an insufficient consideration of the intricate processes involved on a large scale, granular research level. In the context of rapid urban expansion and continuously increasing population density, it is particularly important to conduct in-depth research on the relationship between GSs and psychological stress perception for future urban planning and construction. This study, focusing on the central urban area of Shanghai, utilizes SVI and advanced machine learning methods to reveal the nonlinear impact of GS exposure on residents' psychological stress perception. We adopted a multidimensional stress perception scale that encompasses five independent factors and 12 specific questions, achieving precise quantification of residents' psychological stress perception. By integrating SVM machine learning algorithms and extensive urban street view data, we have successfully developed a novel urban stress perception measurement framework that demonstrates superior performance in stability and explanatory power. Moreover, this framework can be conveniently applied to measure the psychological stress perception of residents in different cities.

This study indicates that GS exposure in central urban Shanghai is generally low, averaging only 0.24, and exhibits significant heterogeneity in spatial distribution. Through spatial correlation analysis, we confirmed a negative effect of GS exposure on psychological stress perception, a relationship that remains stable after controlling for spatial effects. Additionally, we identified a nonlinear characteristic in the alleviating effect of GS exposure on psychological stress, indicating the presence of a threshold. Between 0 and 0.20 GS exposure, increasing such exposure significantly mitigates psychological stress. However, beyond 0.35, the contribution of additional GS exposure to stress relief approaches zero. Analyzing against the stress perception scale, we explored the impact of GS exposure on various stress perception factors, uncovering that it mainly alleviates psychological stress by enhancing residents' sense of relaxation and willingness to stay. This finding provides important guiding principles for future urban planning: when designing GSs, consideration should be given to their impact on residents' psychological comfort and lingering behavior, creating a more pleasant and mentally healthy urban environment.

In conclusion, the findings of this study contribute to the targeted identification of urban environments and guide the precise implementation of policies aimed at enhancing urban GSs. To better understand the causal relationship between GS exposure and mental health, we recommend using longitudinal data in future research to track the dynamics of green exposure and its impact on psychological stress perception. Additionally, future studies should conduct comparative research across different countries, regions, and cities, and analyze the results within more complex social, economic, and cultural contexts. We suggest that future urban planning and construction fully consider the threshold effect of GS exposure, promoting not only the quantity of GSs but also their strategic layout and quality to optimize their mental health benefits. Establishing mechanisms for the regular monitoring and evaluation of the health impacts of existing and new GSs is crucial. Such data should inform ongoing urban planning decisions and help refine GS policies to maximize public health outcomes. We advocate for enhanced collaboration among disciplines such as psychology, urban planning, and environmental science to jointly explore comprehensive solutions for alleviating psychological stress among urban residents and improving their quality of life.

## Data Availability

The datasets presented in this study can be found in online repositories. The names of the repository/repositories and accession number(s) can be found below: https://github.com/Ericzzz66/ZTLUrbanStudies/Changlifang_GSexposure_StressPerception.
